# 

**Published:** 1898-08-20

**Authors:** 


**Notices of Births, Deaths, and Marriages are inserted in this column at a charge of 2s. 6d. for an announcement not exceeding four lines.**

### NOTICE TO CORRESPONDENTS.

All MS., Letters, Books for Review, and other matters intended for the Editor should be addressed The Editor, "The Hospital," The Hospital Building, 28 & 29, Southampton Street, Strand, W.C.

The Editor cannot undertake to return rejected MS., even when accompanied by stamped directed envelope.

**Notice to Advertisers and Subscribers.**

All Advertisements, Orders for Copies of The Hospital, and Business Communications should be addressed to **THE MANAGER, (Not to The Editor).** The Hospital, The Hospital Building, 28 & 29, Southampton Street, Strand, London, W.C.

The Cost of Subscription, post free, is as follows:—Per week, 2½d. per quarter, 2s. 8d.; per half-year, 5s. 3d.; per annum, 10s. 6d. Foreign:—Per annum, 15s. 2d.; payable, in all cases, in advance.

**NOTICE.—Vol. XXIII. of "The Hospital" (October, 1897, to March, 1898), in handsome cloth binding, is now on sale at the Office of this Journal, price 7s. 6d. Cases for binding the Numbers contained in this Volume, 1s. 6d. Index to Vol. XXIII., 2½d., post free.**

**The Monthly Part for September will be ready on September 1. Price 6d., by post 8d.**

### The Student of Medicine.

### APPOINTMENTS.

J. R. Benson, F.R.C.S.Eng., appointed Sambrooke Surgical Registrar and Tutor in Surgery at King's College Hospital. Dr. Cuffe, appointed Medical Officer for the Kilsallaghan Dispensary District. W. Duncan, M.B., C.M.Glasg., appointed Medical Officer of Health for Clay Cross Urban District. H. W. Haydon, M.R.C.S.Eng., L.R.C.P.Lond., appointed Medical Officer for the Wade District of the Bodmin Union. T. Jones, M.R.C.S., L.S.A., appointed Medical Officer for the Middle District of the Whitechapel Union. O. M. S. Lewin, M.B.Lond., appointed Assistant Medical Officer to the Chorlton Union Workhouse. G. Master, M.B., B.C.Cantab., appointed Assistant Medical Officer to the Suffolk General Hospital. E. E. Norton, M.R.C.S. L.R.C.P.Lond., D.P.H., appointed Medical Officer of the Workhouse and Infirmary of the Parish of St. Leonard, Shoreditch. H. W. Oulton, M.D., appointed Surgeon to the Dublin Metropolitan Police Force. H. B. W. Plummer, M.R.C.S, M.D., appointed Honorary Surgeon to the West Bromwich District Hospital. A. S. St. John, M.R.C.S., L.R.C.P.Lond., appointed Medical Officer for the Eltham District of the Lewisham Union. P. C. E. Tribe, M.B.Lond., L.R.C.P., appointed Sambrooke Medical Registrar and Tutor in Medicine at King's College Hospital. H. E. White, M.R.C.S., L.R.C.P., appointed Assistant Medical Officer to the Parish of Birmingham Infirmary. J. Williamson, M.R.C.S., L.R.C.P., appointed House Physician at the General Infirmary, Leeds. G. Wilkinson, B.A., M.B.Cantab., F.R.C.S.Eng., appointed Honorary Surgeon to the Royal Hospital, Sheffield.

JUST OUT. Price 1/.

**THE EFFECTS OF THE DIAMOND JUBILEE ON THE RESOURCES OF THE VOLUNTARY CHARITIES.**

**THE VOLUME OF CHARITY. IS THERE A MAXIMUM YIELD?**

By SIR HENRY BURDETT, K.C.B.

London: The Scientific Press, Ltd., 28 & 29, Southampton Street, Strand, W.C.

